# Ablation of Runx2 in Ameloblasts Suppresses Enamel Maturation in Tooth Development

**DOI:** 10.1038/s41598-018-27873-5

**Published:** 2018-06-25

**Authors:** Qing Chu, Yan Gao, Xianhua Gao, Zhiheng Dong, Wenying Song, Zhenzhen Xu, Lili Xiang, Yumin Wang, Li Zhang, Mingyu Li, Yuguang Gao

**Affiliations:** 1 Department of Pediatrics and Preventive Dentistry, Hospital Affiliated to Binzhou Medical University, Binzhou, 256600 Shandong China; 2 Institute of Stomatology, Binzhou Medical University, Yantai, 255000 Shandong China; 3 Shanghai Key Laboratory of Stomatology, Shanghai Research Institute of Stomatology, Ninth People’s Hospital, Faculty of Medicine, Shanghai Jiao Tong University, Shanghai, 200011 China

## Abstract

Runt-related transcription factor 2 (Runx2) is involved in the early stage of tooth development. However, only few studies have reported the role of Runx2 in enamel development, which may be attributed to that *Runx2* full knockout mice cannot survive after birth. In the present study, we successfully established a Runx2*-*deficient mouse model using a conditional knockout (cKO) method. We observed a significant reduction in the degree of mineralization and the decreased size of enamel rods in cKO mice. Histological analysis showed the retained enamel proteins in enamel layer at maturation stage in cKO molars. Further analysis by qRT-PCR revealed that the expressions of genes encoding enamel structure proteins, such as amelogenin (AMELX), ameloblastin (AMBN) and enamelin (ENAM), were increased in cKO enamel organs. On the other hand, the expression of kallikrein-related peptidase-4 (KLK4) at the mRNA and protein levels was dramatically decreased from late secretory stage to maturation stage in cKO enamel organs, while the expression of matrix metalloproteinase-20 (MMP-20) was not significantly altered. Finally, immunohistochemistry indicated that the uptake of amelogenins by ameloblasts was significantly decreased in cKO mice. Taken together, Runx2 played critical roles in controlling enamel maturation by increasing synthesis of KLK4 and decreasing synthesis of AMELX, AMBN and ENAM.

## Introduction

Tooth enamel, which covers the tooth crown, has the highest degree of mineralization in the body. Ameloblasts (am) secrete matrix proteins during enamel development, while these proteins are completely removed when enamel achieves its final hardened form. Mature enamel only contains less than 1% organic materials by weight, while other calcified tissues, such as bone and cartilage, consist of approximately 20–30% organic components^1^. Therefore, the process of enamel biomineralization is different from general biomineralization of calcified tissues.

Enamel development consists of two major stages: secretory and maturation stages^2^. During the secretory stage, columnar-shaped ameloblasts secrete enamel proteins, such as amelogenin (AMELX), ameloblastin (AMBN) and enamelin (ENAM), into the enamel matrix (em), which act as scaffolds for enamel crystallization. The most abundant matrix protein is AMELX that accounts for about 90% of the total matrix proteins of immature enamel, and the remaining 10% consists of AMBN and ENAM^3,4^. Matrix metalloproteinase-20 (MMP-20), which is expressed in ameloblasts at the secretory stage, processes enamel proteins at secretory surfaces of ameloblast distal membranes to facilitate crystallite elongation^5,6^. Aberrations in appositional growth of enamel crystallites during the secretory stage cause hypoplastic amelogenesis imperfecta (AI), which is characterized by thin but mineralized enamel. Mutations in *AMELX*, *ENAM*, *AMBN* and *MMP-20* genes cause hypoplastic AI^7–14^. Once enamel attains its full thickness, enamel development progresses to the maturation stage, and ameloblasts shrink in size and greatly reduce their expressions of enamel proteins. During the maturation stage of enamel development, all enamel proteins undergo enzymatic degradation by kallikrein-related peptidase-4 (KLK4) to facilitate enamel mineralization^15,16^. Minerals are deposited on the sides of fully elongated crystallites, resulting in increased width and thickness. Delayed removal of enamel proteins may cause hypomaturation AI due to defective enamel biomineralization^17^. Genetic studies in human and mice confirm that KLK4 is critical for proper maturation of enamel crystals^18,19^. Collectively, the process of enamel development requires tight control of both spatial and temporal expressions of numerous genes. Therefore, we hypothesized that such highly specialized developmental process was regulated by an ameloblast-specific mechanism.

As a critical transcription factor, runt-related transcription factor 2 (Runx2) is a critical transcription factor regulating bone formation. Bone tissue contains hydroxyapatite crystals and several types of extracellular matrix (ECM) proteins, including type I collagen, bone sialoprotein, osteonectin, osteopontin and osteocalcin^20,21^. These genes are regulated via Runx2 binding sites in the proximal promoter of the respective genes^22^. Genetic studies on Runx2 deficiency using traditional knockout mice have shown a complete lack of ossification^23^ and arrested tooth formation at the cap stage^24^. In humans, genetic disorder of Runx2 has been reported to cause cleidocranial dysplasia (CCD) and dental anomalies^25^. These genetic findings highlight the critical roles of Runx2 in the development of mineralized tissues, but its role in enamel development remains largely unexplored. It has been reported that Runx2 is expressed in ameloblasts during the late secretory and maturation stages^26^. In the present study, we aimed to investigate the role of Runx2 in enamel development. A conditional knockout (cKO) mouse model was successfully established, and we found that ameloblast-specific ablation of Runx2 in mice resulted in enamel hypomaturation phenotypes. Furthermore, the expression level of *KLK4* gene was significantly decreased in cKO mice. Our findings revealed the biological functions of Runx2 in amelogenesis and provided insights into the molecular mechanisms underlying enamel maturation.

## Results

### Validation of Runx2 ablation in teeth of cKO mice

**Figure 1:**
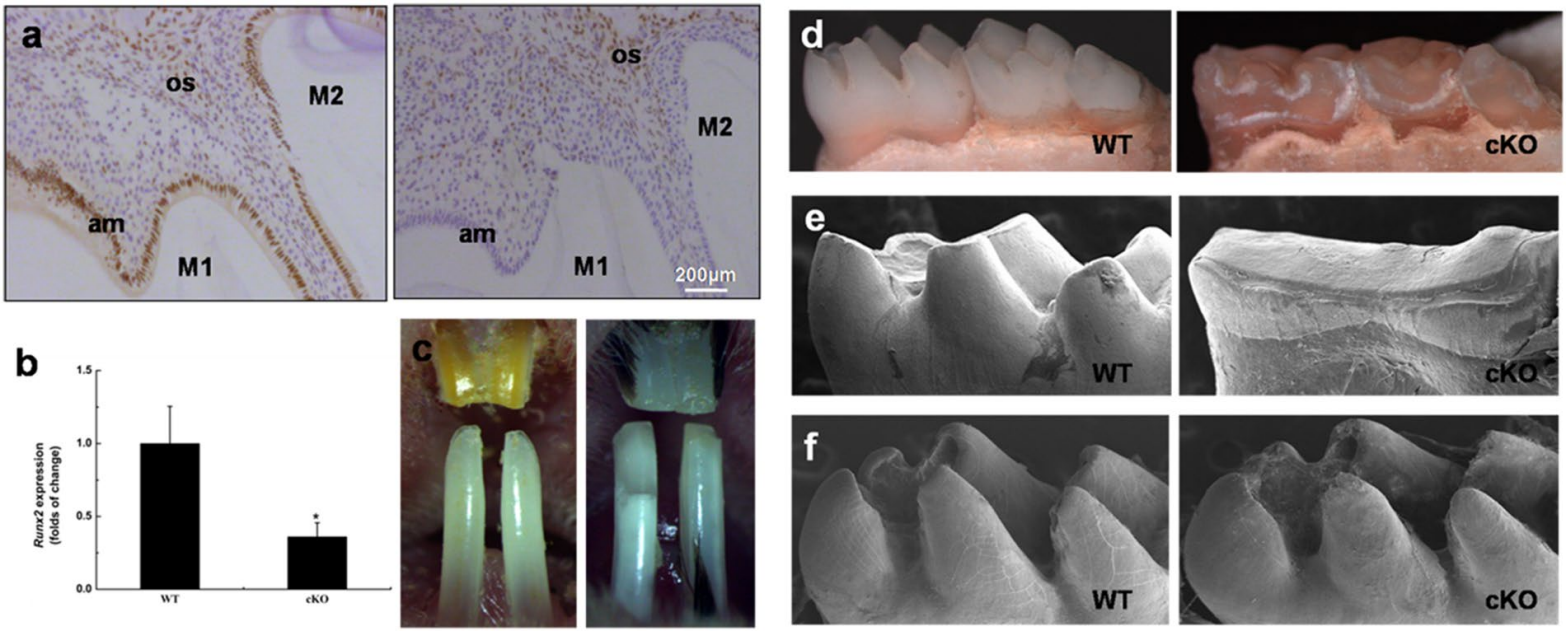
Inactivation of *Runx2* in ameloblasts and associated enamel defects. Panel a, validation of *Runx2* inactivation in PN10 (postnatal day-10) mice by IHC. There was no Runx2 to be detected in the cKO ameloblasts (right), while the WT ameloblasts showed a positive staining (left). Panel b, qRT-PCR analysis of mandibular molars in PN10 mice to observe *Runx2* expression. The reduction of *Runx2* expression in the cKO molars was confirmed. Panel c, frontal view of maxillary and mandibular incisors in 3-month-old mice. Incisors of cKO mice (right) showed a chalk-white appearance and chipped incisal edges as compared with WT incisors (left). Panel d, severe attrition was observed in cKO mandibular molars of 3-month-old mice. Panel e, lingual view of the first molars from 6-month-old mice by SEM demonstrated occlusal wear to the level of dentin in cKO molars. Panel f, lingual view of the first molars from PN14 mice by SEM. These images showed the molars prior to eruption; the sizes of the tooth crowns and the thickness of the enamel appeared to be similar in the two genotypes.

Loss of Runx2 protein was confirmed by IHC staining using a polyclonal antibody against Runx2. Specifically, the lower first molars of WT mice showed positive staining for Runx2 in ameloblasts and surrounding bone tissues, while no positive Runx2 staining was observed from ameloblasts in the first lower molars of cKO littermates, but its positive staining was detected in surrounding bone tissues (Fig. 1a). Moreover, we further confirmed that the *Runx2* expression at the mRNA level was significantly decreased in cKO mice (Fig. 1b). These findings indicated that the cKO mouse model was successfully established.

### Severe attrition in cKO mice

Compared with WT mice, the cKO mice showed no obvious differences in tooth eruption. However, at the gross level, the incisors of 3-month-old cKO mice (when teeth may be affected by mechanical stresses associated with function) exhibited a chalky-white labial enamel, considerable attrition and enamel flaking at the incisor tips, while WT littermates had pointed sharp incisors with a yellow-brown color and transparent glossy appearance (Fig. 1c).

Examination of the molars in 3-month-old mice by stereoscopic microscopy revealed that WT mice had prominent and pointed cusps on all three molars, while the molars of cKO mice were excessively abraded with flattened cuspal surfaces (Fig. 1d). Such severe attrition of molar cusps in 6-month-old cKO mice became highly evident in the scanning electron micrographs, displaying flattened and heavily worn molar cusps (Fig. 1e), which was not the case for the postnatal day-14 (PN14) cKO molars (immediately preceding eruption) that exhibited relatively normal crown size and shape (Fig. 1f). These findings suggested that the phenotypic changes in the enamel of cKO mice might be caused by hypomaturation but not hypoplasticity.

### Severely affected enamel mineralization in cKO mice

**Figure 2:**
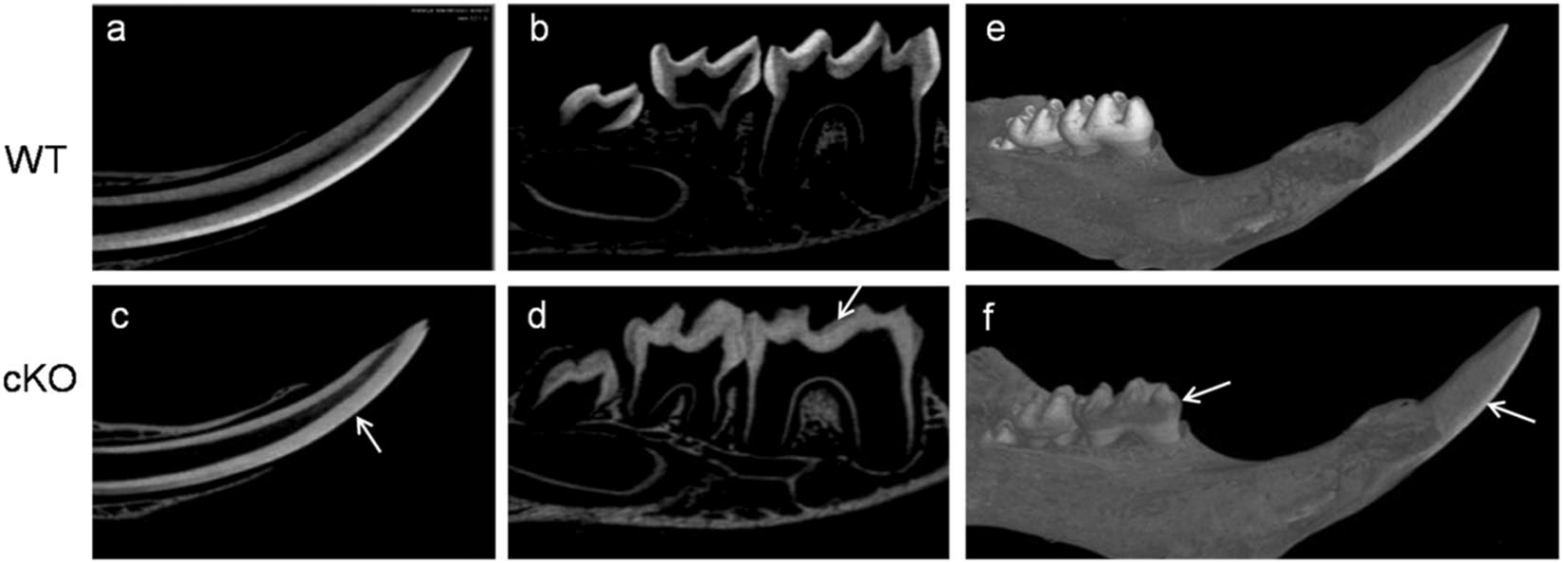
µCT analysis of the mandibles from WT and cKO mice at 3-week-old of age. The enamel of incisors (**a**) and molars (**b**) in WT mice showed a sharp contrast with dentin, while the incisors (**c**) and molars (**d**) in cKO mice displayed a dramatic reduction in enamel mineral densities (arrows). Three-dimensional reconstruction of the mandibles further showed the decreased mineral density of enamel in molars and incisors (arrows) in cKO mice (**f**) as compared with WT mice (**e**).

To further clarify the enamel hypomaturation defects of cKO mice, we performed µCT analyses to examine the enamel mineralization in cKO mice. The mineral density of enamel layers of WT mice showed contrast with dentin (de). However, the mineral density of enamel layers of cKO mice was low compared with those of the control mice, showing weak contrast with dentin, and the mineral density of enamel in incisors and molars was significantly decreased in cKO mice (Fig. 2a–d). Three-dimensional reconstruction showed that the enamel exhibited a low mineral density in cKO mice (Fig. 2e,f). These findings demonstrated that Runx2 deficiency caused a pronounced delay in enamel mineralization.

### Enamel structure defects in cKO mice

**Figure 3:**
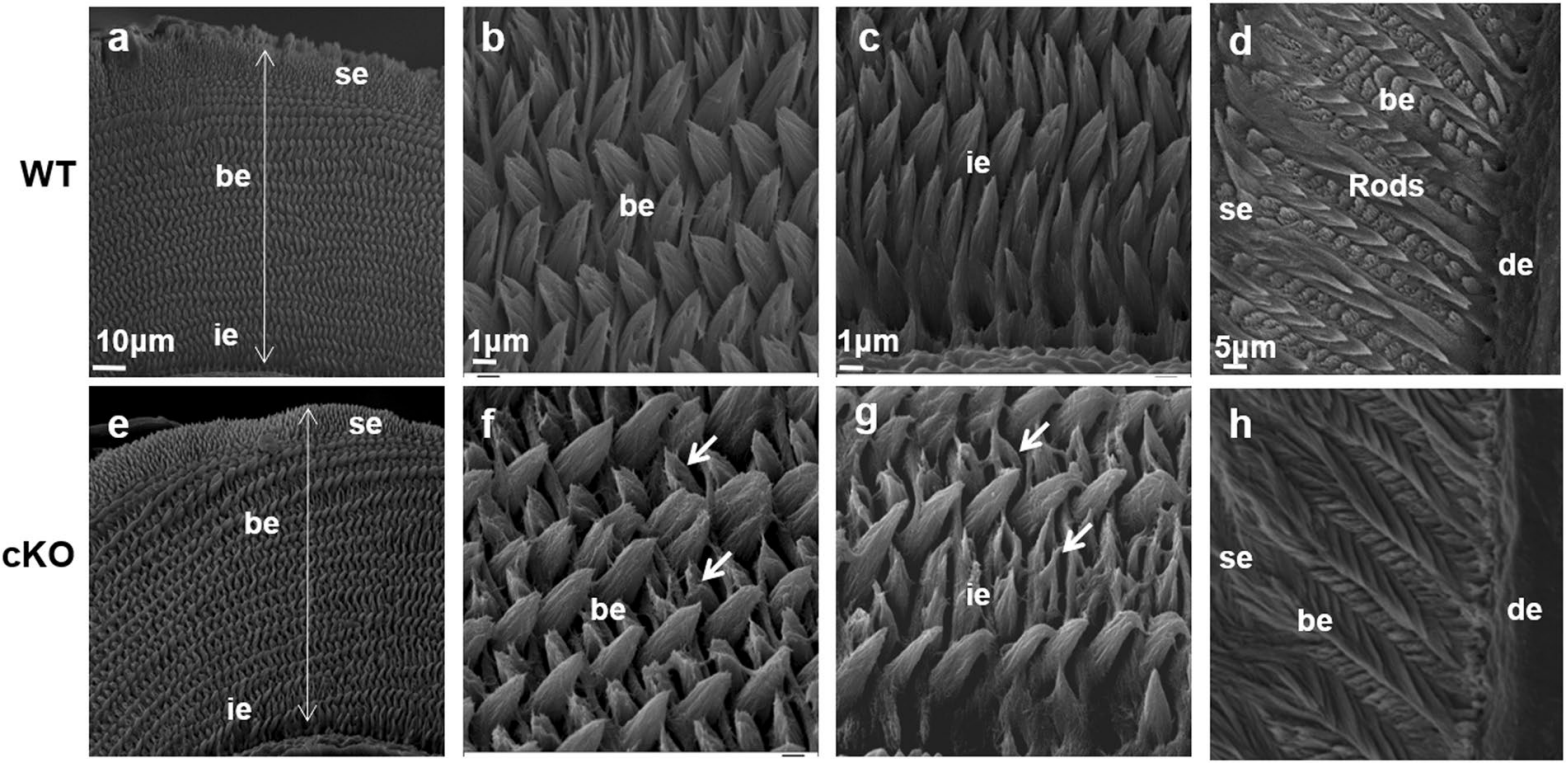
SEM analysis of the ground sections of enamel from 3-week-old mice. Sections from WT incisor (**a**–**c**) and molar (**d**) are shown in the upper panel. Comparable sections from cKO incisor (**e**–**g**) and molar (**h**) are shown in the lower panel. The sections showed decussating enamel rods in WT and cKO mice (**a**,**b**), and there was no obvious difference in enamel thickness (double-headed arrows). High magnification of the ground sections of incisors revealed that crystallites in the bulk enamel (be) and inner enamel (ie) in cKO mice were less impacted, and enamel rods were thin and disorganized (arrowheads) (**f**,**g**) as compared with WT mice (**b**,**c**). The coronal-sections of mandibular molars in WT mice showed enamel rods clearly (**d**), but the enamel rods in the sections of cKO mice were not exposed adequately (**h**).

To analyze enamel hypomineralization defects of cKO mice, we examined the enamel structure of incisors and molars by SEM (Fig. 3). Decussating enamel rods were observed in incisors of WT and cKO mice, and the enamel thickness was not significantly altered (Fig. 3a,e). The enamel of WT incisors showed solid structure in rods and interrods (Fig. 3b,c), while the enamel rods of cKO incisors were thin and disorganized (Fig. 3f,g). The enamel rods in the coronal sections of the first mandibular molars were not adequately exposed in cKO mice (h) compared with WT mice (d). These findings suggested that Runx2 deficiency in ameloblasts impaired the expanding of enamel crystals, leading to delayed removal of proteins from enamel matrix.

### Excessive enamel proteins in cKO mice

**Figure 4:**
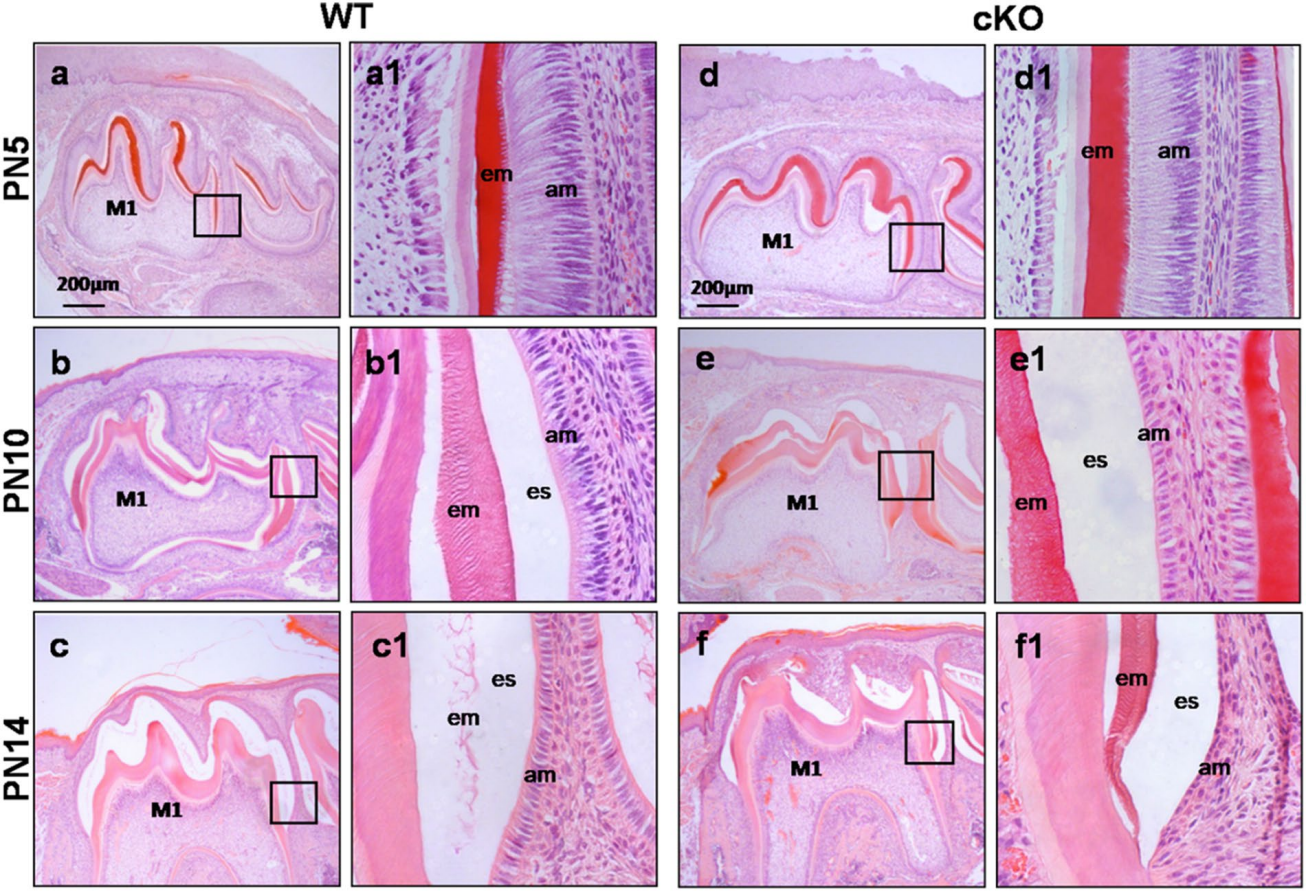
Histological analysis of the mandibular molars of WT and cKO mice. Low-magnification views of the PN5, PN10 and PN14 mandibular molars (**a**–**f**). Higher magnification views of the square regions were in the right column to detail ameloblasts and organic matrix (a1–f1). In PN5 mice, enamel matrices were actively being secreted by ameloblasts and showed eosinophilic staining (**a**,**d**). No evident histological differences were identified between the first molars of the two genotypes (a1,d1). In PN10 cKO mice, more matrix proteins were retained near the cusp tips where enamel maturation first commenced (**b**,**e**), and ameloblasts appeared shorter in height (b1,e1). By PN14 (immediately prior to eruption), the enamel proteins were absent in WT molars, while cKO molars retained eosinophilic materials in enamel, and ameloblasts were completely depolarized (c,c1,f,f1).

To investigate the causes of enamel defects in cKO mice, we analyzed the histology of developing mandibular molars at PN5, PN10 and PN14. Ameloblasts in the first molars during these periods were at late secretory (PN5), mid-maturation (PN10) and late-maturation (PN14) stages, respectively. Ameloblasts at the late secretory stage exhibited a typical tall columnar morphology in WT and cKO mice, and eosinophilic enamel matrices appeared to be similar (Fig. 4a,a1,d,d1), suggesting that Runx2 deficiency did not significantly affect ameloblasts at the secretory stage. The enamel layers near the cusp tips of the cKO first molars at the mid-maturation stage showed more matrix proteins (Fig. 4e), and ameloblasts were obviously shortened (Fig. 4e1) compared with WT mice (Fig. 4b,b1). In cKO enamel organs at the late-maturation stage, ameloblasts completely lost polarization, and residual enamel proteins in the enamel at cervical area were abundant (Fig. 4f,f1), whereas only a small amount of enamel proteins was found in the enamel of WT first molars (Fig. 4c,c1). These results demonstrated that Runx2 deficiency in ameloblasts resulted in the accumulation of matrix proteins during the maturation stage of amelogenesis.

### Increased expressions of *AMELX, AMBN and ENAM*, and decreased expression of *KLK4* in cKO mice

**Figure 5:**
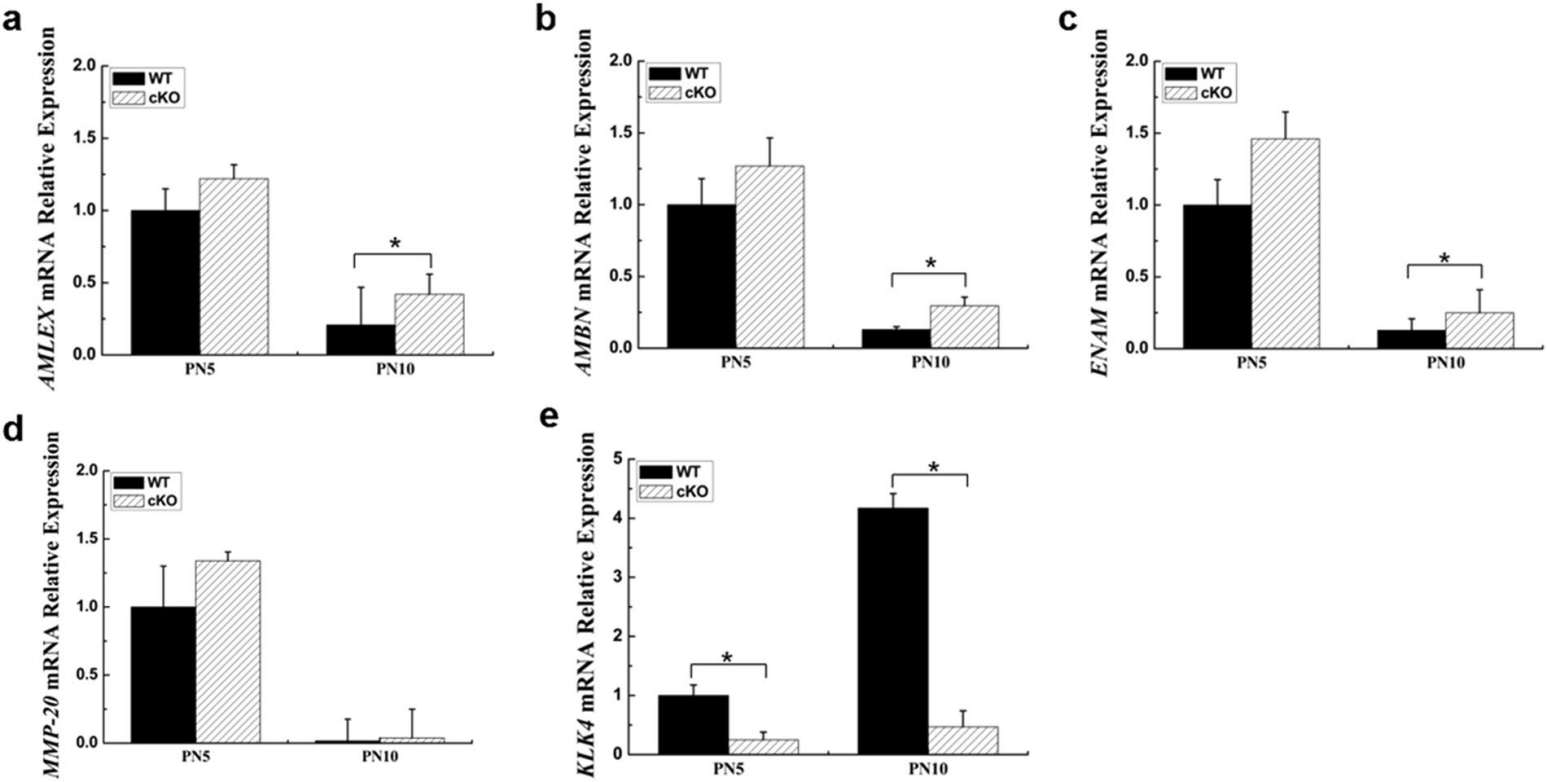
Expressions of selected enamel genes were analyzed by qRT-PCR. At PN5, the expressions of genes, such as *AMLEX*, *AMBN* and *ENAM* were slightly elevated in cKO enamel organs. At PN10, the expressions of these genes were significantly up-regulated in cKO enamel organs as compared with WT enamel organs (**a**–**c**). Expression of *MMP-20* gene in PN5 or PN10 cKO enamel organs was not significantly altered as compared with WT enamel organs (**d**). Expression of *KLK4* gene was significantly down-regulated in both PN5 and PN10 cKO enamel organs (**e**). *p < 0.05

In the present study, we performed qRT-PCR analysis to identify the ameloblast-specific gene expression patterns associated with the hypomineralized enamel in cKO mice, specifically for genes encoding enamel structure proteins and enamel processing proteins (Fig. 5). The expression levels of *AMELX, AMBN* and *ENAM* were slightly increased in PN5 cKO enamel organs, whereas their expressions were significantly increased in PN10 cKO enamel organs (Fig. 5a–c). For the enamel processing proteins, the expression of *MMP-20* was not obviously affected by Runx2 deficiency in PN5 and PN10 mice (Fig. 5d), but the *KLK4* expression at the mRNA level was dramatically decreased in PN5 cKO enamel organs and further reduced in PN10 cKO enamel organs (Fig. 5e). These results suggested that enamel proteins were retained in enamel matrix of cKO mice mainly due to the reduced *KLK4* expression, which was also correlated with the elevated expressions of *AMELX, AMBN* and *ENAM* to some extent.

### Decreased secreted protease KLK4 in ameloblasts of cKO mice

**Figure 6:**
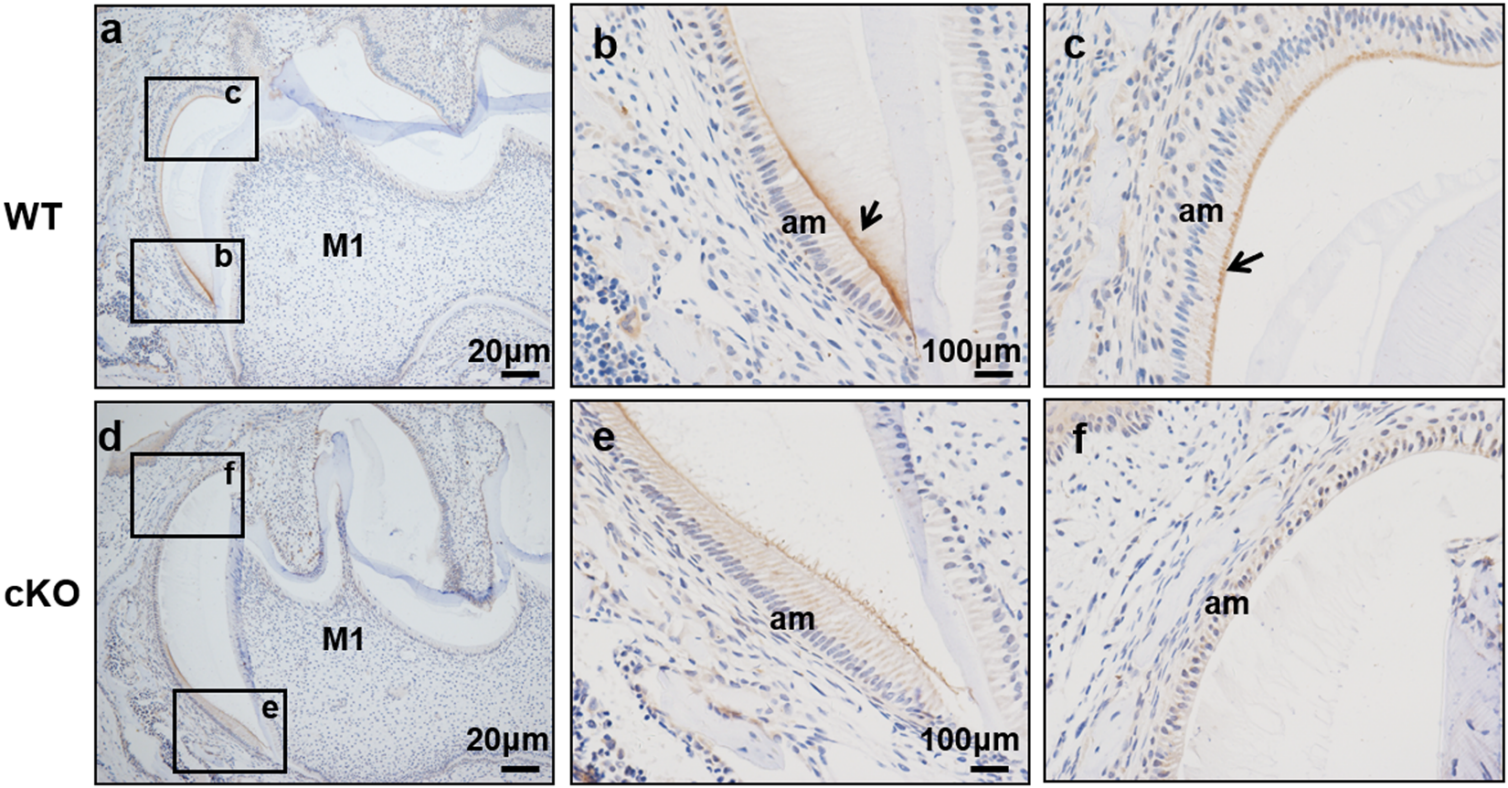
IHC analysis of enamel organs in the mandibular molars of WT and cKO mice at PN10. Sagittal sections of mandibular molars were stained with KLK4 antibody and observed at low magnification (**a**,**d**). Higher magnification images of the square regions were shown in the right column (**b**,**c**,**e**,**f**). At PN10 mice, the first molar (M1) enamel development was found in the late secretory stage near the cervical loop and in the mature stage of enamel near the tooth tip, the enamel matrix was mostly degraded from cervical loop to tooth tip, the main distribution was at the distal end of the ameloblasts in PN10 WT mice (as arrowhead showed in b and c), but in cKO mice ameloblasts, no obvious staining was detected.

In order to further verify the expression of KLK4 at the protein level in cKO mice, we conducted IHC using antibody against KLK4. We found that in PN10 WT mice, the staining was strongly detected in the distal end of ameloblasts. However, in cKO mice, the staining was not very obvious (Fig. 6). This result was consist with qRT-PCR result, confirming that the ablation of Runx2 might cause down-regulated expression of KLK4 at the protein level.

### Decreased intracellular AMELX proteins in ameloblasts of cKO mice

**Figure 7:**
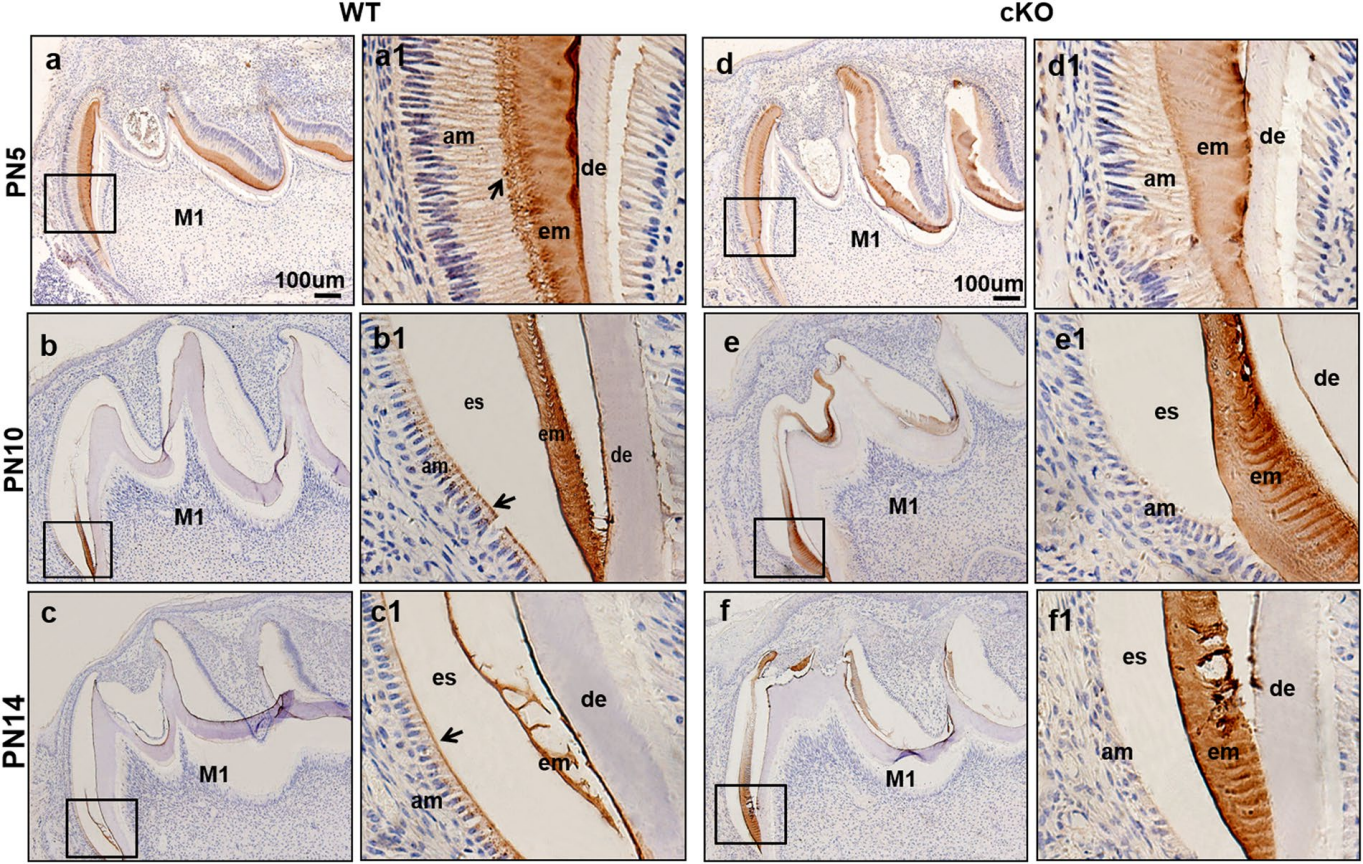
IHC analysis of enamel organs in the mandibular molars of WT and cKO mice at PN5, PN10 and PN14. Sagittal sections of mandibular molars were stained with AMELX antibody and observed at low magnification (**a**–**f**). Higher magnification images of the square regions were shown in the right column (a1–f1). In PN5 WT mice, AMELX was detected in the enamel matrix (**a**), and the Tomes’ processes in secretory ameloblasts (arrowhead) (a1). As enamel progressed to maturation, the early maturation-stage ameloblasts at the center and apical regions were intensely stained in small puncta (arrowhead) (**b**, b1); when most enamel proteins were reabsorbed at the mid-maturation stage, AMELX staining was decreased in the cytoplasma and apical membranes of ameloblasts (arrowhead) (**c**,c1). In cKO mice, the staining persisted in enamel matrix (**d**–**f**), but no obvious staining was detected in cKO ameloblasts (d1–f1).

Because AMELX is the predominant ECM protein and the cKO mice showed retained matrix proteins in their enamel, we conducted AMELX staining in WT and cKO molars (Fig. 7). In WT mice, AMELX was strongly detected in the enamel matrix from the secretory stage to the maturation stage of enamel organs (Fig. 7a–c). Under high magnification, the staining was intense at Tomes’ processes of ameloblasts (Fig. 7a1), and it was persisted in the distal membranes and cytoplasm of ameloblasts at the mid-maturation and late-maturation stages (Fig. 7b1,c1). In cKO mice, the staining was strongly detected in the enamel matrix (d-f), while no obvious staining was observed in ameloblasts of cKO mice (d1–f1). Combined with the qRT-PCR findings that the *KLK4* expression was dramatically decreased in cKO mice, these results suggested that the decreased uptake of AMELX protein by ameloblasts from enamel matrix was related to the reduced degradation of enamel proteins by KLK4 in cKO mice.

## Discussion

To clarify the function of Runx2 in enamel development, we successfully generated the cKO mice with loss of *Runx2* gene in ameloblasts.

The ablation of Runx2 in cKO mice was confirmed by IHC and qRT-PCR analyses. Ameloblasts in cKO mice exhibited non-specific staining using an anti-Runx2 antibody, whereas specific and intense staining was observed in the ameloblasts of WT mice. Similarly, enamel organs at the maturation stage in cKO mice showed decreased *Runx2* expression at the mRNA level compared with WT mice. These initial characterizations indicated that *Runx2* was functionally knocked out in ameloblasts of our cKO mice, which exhibited significant enamel hypomineralization defects and abnormal morphology of ameloblasts in unerupted teeth.

As a key transcription factor, Runx2 plays a critical role in bone development. Mutations in the *RUNX2* gene cause the CCD, an autosomal dominant disorder in humans characterized by defective bone formation^27^. CCD also results in dental defects, including enamel hypomineralization^28,29^. Our findings showed that Runx2 deficiency in ameloblasts resulted in enamel hypomineralization, which were consistent with the enamel defects in CCD patients.

More than 60% of mineral deposition on enamel surface occurs during the maturation stage of enamel development, where enamel crystals expand in width and thickness by mineral deposition^30^. In our study, the cKO enamel showed decussating enamel rods, and the thickness of enamel was not altered. However, the arrested growth of enamel rods and the retained organic matrices in the cKO enamel suggested the impairment of enamel biomineralization at the maturation stage.

During the secretory stage of enamel development, ameloblasts in cKO mice displayed the high columnar cell morphology. During the maturation stage, the cKO ameloblasts showed an abnormal morphology, and the cKO enamel showed excessive matrix proteins. Based on the observed histological differences between WT and cKO enamels, we speculated that Runx2 regulated the protein accumulation in enamel matrix by targeting either genes encoding enamel structural proteins (such as AMELX, ENAM and AMBN) or those encoding enamel matrix processing proteases (such as MMP-20 and KLK4). Runx2 can regulate bone development by activating or inhibiting the expressions of target genes^31^. To correlate Runx2 and the ameloblast-specific downstream targets, we analyzed the expressions of enamel matrix protein genes at the mRNA level by qRT-PCR. Our data revealed that the expressions of *AMELX*, *AMBN* and *ENAM* were elevated in maturation stage in cKO enamel organs, suggesting that the enamel hypomaturation in cKO mice was triggered by the elevated expressions of enamel proteins at the maturation stage. Currently, it remains unknown why the expressions of AMLEX, AMBN and ENAM at the mRNA level were higher in cKO mice. It has been reported that Runx2 down-regulates the expressions of Runx1, Ptgs2 and Tnfaip6 in luteinizing granulosa cells as a transcription repressor to control follicular development^32^. Therefore, it was possible that Runx2 down-regulated the expressions of enamel matrix protein genes as a transcription repressor during enamel development. Among the genes encoding enamel matrix processing proteases, the expression of *KLK4* at the mRNA and protein levels was dramatically decreased in cKO enamel organs during the late secretory stage and maturation stage. This finding was consistent with the cKO mice resembling *KLK4* null phenotypes. *KLK4* null mice display hypomaturation enamel defects with normal enamel thickness but rapidly abraded enamel following weaning^19^. During enamel development, Runx2 and KLK4 are both expressed at maturation stage of ameloblasts^19,33^. Therefore, Runx2 might up-regulate the KLK4 expression as a transcriptional activator to control enamel maturation, and further studies are needed to clarify this hypothesis.

In *KLK4* null mice, the molars show residual proteins throughout the enamel layer near tooth eruption^19^, suggesting that reabsorption of enamel matrix protein is severely affected. AMELX, which constitutes the majority of enamel matrix proteins, is secreted at the early stage of enamel development and subsequently hydrolyzed and reabsorbed by ameloblasts at the maturation stage^34,35^. As AMELX is removed, calcium is transported through ameloblasts and deposited along with phosphate to form the mineralized enamel matrix^36^. We found that the Runx2-deficient ameloblasts had less intracellular AMELX, although the expression of *AMELX* at the mRNA level was increased at the maturation stage. This finding suggested that Runx2 deficiency in ameloblasts delayed reabsorption of enamel proteins, which might be correlated with the decreased *KLK4* expression in cKO mice.

In conclusion, Runx2 played an essential role in controlling enamel maturation by suppressing the synthesis of enamel structure proteins, such as AMELX, AMBN and ENAM, and it was also involved in promoting the synthesis of KLK4 to degrade enamel matrix proteins. Our findings enriched our knowledge of enamel maturation. We speculated that Runx2 interacted with its target gene to regulate the expressions of related genes, consequently affecting enamel biomineralization. Therefore, it is necessary to perform further studies in order to explore the specific regulatory mechanisms of Runx2 in enamel development.

## Materials and Methods

All animal protocols were reviewed and approved by the Ethical Committee of the Institute of Zoology, Chinese Academy of Sciences. All efforts were made to minimize the suffering of animals according to recommendations proposed by the European Commission (1997) and carried out in accordance with the approved protocol. This article did not contain any studies with human participants performed by any of the authors.

### Generation of Runx2-floxed mice

Mice with a *Runx2*-floxed allele were generated by Cyagen Biosciences (Guangdong, China). In the targeting vector, exogenous elements were inserted into introns on both sides of exon 2 of the mouse *Runx2* gene. Introns on both sides of exon 2 were flanked by loxP sequences (Supplemental Fig. 1). LoxP elements were inserted into the non-coding region. Therefore, the insertion of loxP elements into these introns was not expected to interfere with mRNA splicing. When planning the targeting strategy, we considered that removal of exon 2 would result in a frame shift of downstream exons. Introns on both sides of the targeting vector also contained a Neo cassette flanked by two flippase recognition target (FRT) sites. Therefore, a conditional allele could be generated after treatment with the flippase (Flp) recombinase. After introduction of Flp recombinase, the Neo cassette was removed, leaving exon 2 flanked by two loxP sites. Mice with one allele of *Runx2*, which was floxed by two loxP elements containing no Neo cassette, were designated as *Runx2*^*flox/*+^ mice. These *Runx2*^*flox/*+^ mice were inbred to establish *Runx2*^*flox/flox*^ mice.

DNA extracted from the mouse tails was analyzed by polymerase chain reaction (PCR) for genotyping to identify the 5′loxP site from the WT allele using the following set of primers: forward, 5′-GCGCCTTGCTAGACAGACAGGC-3′ (“a” in Supplemental Fig. 1a); and reverse, 5′-CCATCCATCACCAAGACCCTGC-3′ (“b” in Supplemental Fig. 1a). PCR with these primers yielded a product of 304 base pairs (bp) for *Runx2*^*flox/flox*^ allele and a 243-bp fragment for the wild-type allele (Supplemental Fig. 1b). PCR was used to identify the Frt and 3′loxP site using set of primers: forward, 5′-GGGGGTTTAGCACGTACAGTAGGG-3′ (“c” in Supplemental Fig. 1a); and reverse, 5′-CTCCAACTGTCCCTGTATGAACGC-3′ (“d” in Supplemental Fig. 1a). PCR with these primers yielded a product of 308 bp for *Runx2*^*flox/flox*^ allele and a 168-bp fragment for the wild-type allele (Supplemental Fig. 1c).

### Generation of *K14-Cre;Runx2*^flox/flox^ mice

*K14-Cre* mice (004782; the Jackson Laboratory), which express Cre recombinase in the oral mucosal epithelium at approximately 10.5 days post-coitum, were mated with *Runx2*^*flox/flox*^ mice to create *K14-Cre*^+^;*Runx2*^*flox/wt*^ mice. The *K14-Cre*^+^;*Runx2*^*flox/wt*^ mice were then bred with *Runx2*^*flox/flox*^ mice to generate *K14-Cre*^+^;*Runx2*^*flox/flox*^ (cKO) mice. DNA samples from mouse tails were genotyped by PCR. PCR primers as a, b, c and d described above (Supplementary Fig. 1a) were used to identify *Runx2*^*flox/flox*^ mice from the offspring. In addition, a set of specific primers for the Cre transgene was adopted to identify Cre recombinase, forward, 5′-TTCCTC AGGAGTGTCTTCGC-3′ (“e” in Supplementary Fig. 1a); reverse, 5′- GTCCATGTCCTTCCTGAAGC-3′ (“f” in Supplementary Fig. 1a). PCR with the Cre-specific primers yielded a 494-bp fragment from mice expressing Cre recombinase, and no PCR product was amplified from mice expressing the (wild type) WT allele (Supplementary Fig. 1d). The mice were maintained in a conventional animal care facility and given free access to standard mouse chow and water. Animals were sacrificed by CO_2_ asphyxiation.

### Histology and immunohistochemistry (IHC)

Mandibles were collected from three control littermates and three Runx2 mutant. The dissected mandibles were fixed, decalcified and embedded by paraffin as previously described^37^. Sagittal sections of paraffin-embedded mandibular molars were prepared and used for hematoxylin-eosin (H&E) staining or IHC. For IHC analysis, sections were incubated with rabbit polyclonal primary antibodies, including anti-Runx2 (1:50 dilution; ab23981; Abcam, Cambridge, MA, USA), anti-AMELX (1:1,200 dilution; ABT260; Merck KGaA, Darmstadt, Germany), and anti-KLK4 (1:500 dilution; ab71234; Abcam, Cambridge, MA, USA), at 4 °C overnight. Bound antibodies were detected using a Vectastain ABC Elite Kit and diaminobenzidine kit (Vector Laboratories, Burlingame, CA, USA) according to the manufacturer’s instructions. Subsequently, the tissue sections were counterstained with Mayer’s hematoxylin and examined by light microscopy (BX53F; Olympus. Tokyo, Japan). Each set of experiments was repeated at least three times.

### Micro-computed tomography (µCT) analysis

Mandibles were dissected from 3-week-old (when teeth may be not affected by mechanical stresses) WT and cKO mice (n = 3 per genotype), symmetrically cut in half, fixed in 4% paraformaldehyde (PFA; pH 7.2), cleaned of soft tissue, washed in phosphate-buffered saline (PBS) for at least 24 h, transferred into 70% ethanol, dehydrated in an ascending series of alcohol, and then dried naturally in air. Morphological analysis and measurement of enamel mineralization of the mandibular teeth were performed using a µCT system (µCT 50; Scanco Medical AG, Bassersdorf, Switzerland), with a nominal isotropic resolution of 5 µm inside a 19-mm diameter scanning vial under conditions of 70 kVp, 114 mA, 8 W, 500 ms integration time and 500 projections per 180°. Threshold values in the µCT evaluation program (version 6.5-2, Scanco, Wayne, PA, USA) were set such that the mineralized enamel appeared as a white high-density solid next to dentin as a transparent object. Three-dimensional images were reconstructed using VGStudio MAX 2.1 software (Volume Graphics, Heidelberg, Germany).

### Scanning electron microscopy (SEM)

For SEM analysis, hemimandibles from WT and cKO mice (n = 3 per genotype) were fixed and then dehydrated in an ascending series of alcohol. Dried samples were embedded in epoxy resin (West System 105 resin; Gougeon Brothers Inc., Bay City, MI, USA) and ground in cross sections to the buccal alveolar crest for incisors and in coronal sections for first molars. Specimens were etched with 37% phosphoric acid for 20 s, air dried, and vacuum coated with gold particles with an ion sputter coater (Quorum, Q150RS, UK). Specimens were observed under a scanning electron microscope (ZEISS EVO LS 15; Carl Zeiss AG, Jena, Germany) at 10 kV.

### Quantitative real-time PCR (qRT-PCR)

The enamel organs of mandibular molars in WT and cKO mice (n = 3 per genotype), were carefully dissected, and the tissues were immediately snap-frozen in liquid nitrogen. Total RNA was extracted from the enamel organs using Trizol Reagent (Invitrogen, Carlsbad, CA, USA) according to the manufacturer’s instructions. Excellent quality of total RNA was judged by UV absorbance data (OD260/280 = 1.8–2.0 and OD260/230 = 2.0–2.2) and agarose electrophoresis (Supplementary Fig. 2). After quantification, equal amounts of total RNA (1 µg) were reversely transcribed into cDNA using an M-MLV RT system (Promega, Madison, WI, USA). Gene expression levels were determined by qRT-PCR using specific primers (Table 1). The optimal conditions and cDNA dilutions were determined for each gene. GAPDH was selected as a housekeeping gene. qRT-PCR experiments were performed using Fast Start DNA Master SYBR Green I (Roche, Neuilly-sur-Seine, France) on a Roche Light Cycler System. All samples were run in quadruplicate for 40 cycles using a Thermal Cycler (x96; Bio-Rad, Hercules, CA, USA). Relative gene expressions were calculated using the 2^−ΔΔCt^ method. Each experiment was performed in triplicate. Data were expressed as mean ± standard deviation (SD). Statistical analyses were performed by Student’s *t*-test. Values of *p* < 0.05 were considered as statistically significant.

**Table 1:** Primer Sequences and PCR Conditions for qRT-PCR Analyses.

Gene	Oligonucleotides	Sizes (bp)	Tm (°C)
**Runx2**			
P1 P2	5′-AGCCAGGTTCAACGATCTGA-3′ 5′-TAGCTCTGTGGTAAGTGGCC-3′	113	65
***AMELX***			
P1 P2	5′-CACCCTTCAGCCTCATCACC-3′ 5′-GTGTTGGGTTGGAGTCATGG-3′	112	60
***AMBN***			
P1 P2	5′-CCAGCAGCCATCCTTGCAGC-3′ 5′-TGCCACCTGCGGCTCATCTG-3′	174	60
***ENAM***			
P1 P2	5′-ACCCAGGAGACCGCCAAACC-3′ 5′-GGGAATGGCTGAGGTGGCTGG-3′	127	60
***MMP-20***			
P1 P2	5′-GGCGAGATGGTGGCAAGAG-3′ 5′-CTGGGAAGAGGCGGTAGTT-3′	166	60
***KLK4***			
P1 P2	5′-GTCAGCAGCCGGATCATACAAGG-3′ 5′-GCACCAAGACTCCCGAGCAGAAA-3′	106	63
***GAPDH***			
P1 P2	5′-TGTGTCCGTCGTGGATCTGA-3′ 5′-TTGCTGTTGAAGTCGCAGGAG-3′	150	65

## Electronic supplementary material

Supplementary Figure
