# Supplementary material for: Ablation of Runx2 in Ameloblasts Suppresses Enamel Maturation in Tooth Development
Source: Sci Rep. 2018 Jun 25;8:9594. doi: 10.1038/s41598-018-27873-5 (PMC6018461; doi:10.1038/s41598-018-27873-5)

Ablation of Runx2 in Ameloblasts Suppresses Enamel Maturation in Tooth Development

Qing Chu[1], Yan Gao[1], Xianhua Gao[1], Zhiheng Dong[1], Wenying Song[1], Zhenzhen Xu[1], Lili Xiang[1], Yumin Wang[2], Li Zhang[2], Mingyu Li[3], Yuguang Gao[1]*

[1]Department of Pediatrics and Preventive Dentistry, Hospital Affiliated to Binzhou Medical University, Binzhou 256600, Shandong, China

[2]Institute ofStomatology, Binzhou Medical University, Yantai 255000, Shandong, China

[3]Shanghai Key Laboratory of Stomatology, Shanghai Research Institute of Stomatology, Ninth People's Hospital, Faculty of Medicine, Shanghai Jiao Tong University, Shanghai 200011, China

***Correspondence to:**

Yuguang Gao, Department of Pediatrics and Preventive Dentistry, Hospital Affiliated to Binzhou Medical University, Binzhou 256600, Shandong, China

Tel.: +8615254308155

E-mail: gaoyuguang@yahoo.com

Supplemental Figure 1. Targeting vector for creating Runx2-defficient mice and genotyping strategy. (a) The mouse *Runx2* gene contains eight exons. The exon 2 was selected as cKO region. In the targeting vector, a Neo cassette was flanked by two FRT sites. Recombination after Flp recombinase excision removed the Neo cassette from the *Runx2* allele. On Cre recombination, exon2 was removed, leading to the loss of function of *Runx2* gene; (b) We used the primer set of a and b to distinguish the 5 ̓loxP site from the WT allele; PCR with these primers produced a 304-bp fragment for the mutant allele

and a 243-bp fragment for WT allele. (c) The primer set of c and d was used to identify FRT and 3′loxP site. Note that the use of primers c and d was expected to generate a 308-bp fragment for the mutant allele and a 168-bp fragment for WT allele. The primer set of e and f was used to identify the *Runx2*-ablated allele in the *K14-Cre;Runx2*$^{flox/+}$ or the *K14-Cre;Runx2*$^{flox/flox}$(cKO) mice; PCR with these primers did not produce any fragment for the *Runx2*-floxed (*Runx2*$^{flox/+}$ and *Runx2*$^{flox/flox}$) or WT alleles, but produced a 494-bp fragment in the *K14-Cre;Runx2*$^{flox/flox}$(cKO) mice.

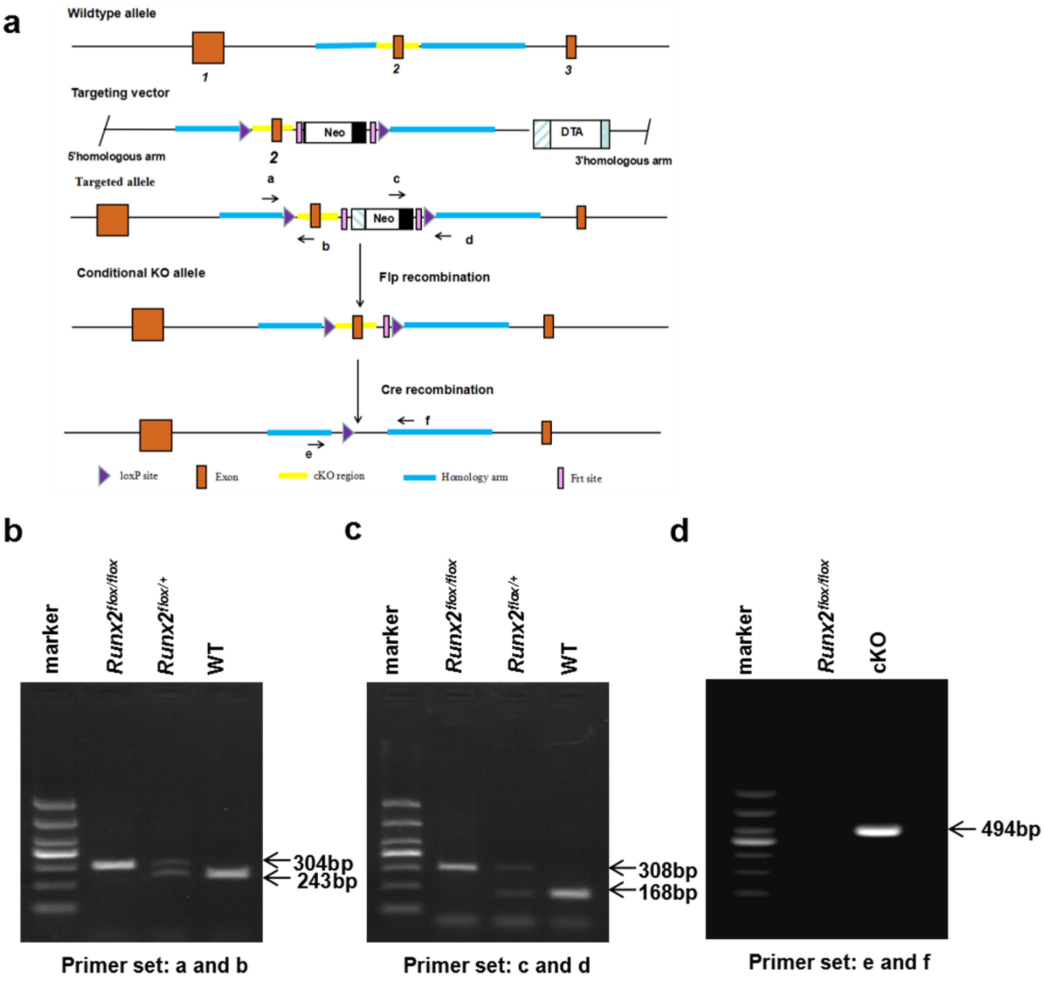

Supplemental Figure 2. Electrophoresis of total RNA on 1.0% agarose gel. Total RNA was extracted from 5-day-old WT, cKO and 10-day-old WT, cKO mice. The electrophoresis results showed that the 28s,18s and 5s bands were clear, and there was no obvious smear between bands. Results indicated that the quality of total RNA in the qRT-PCR was excellent.

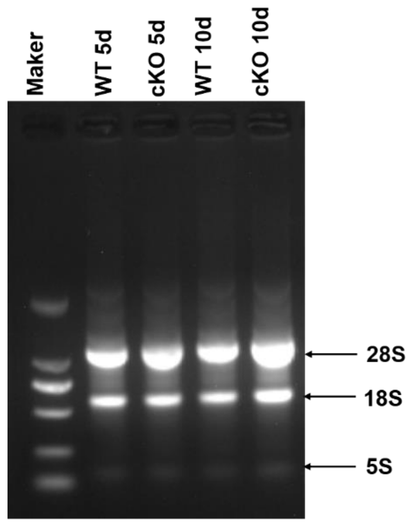

Supplement: Supplementary file 1 — Supplementary Figure [file 41598_2018_27873_MOESM1_ESM.pdf]
